# Correction: Zhang et al. Two SEPALLATA MADS-Box Genes, *SlMBP21* and *SlMADS1*, Have Cooperative Functions Required for Sepal Development in Tomato. *Int. J. Mol*. *Sci*. 2024, *25*, 2489

**DOI:** 10.3390/ijms25158519

**Published:** 2024-08-05

**Authors:** Jianling Zhang, Zongli Hu, Qiaoli Xie, Tingting Dong, Jing Li, Guoping Chen

**Affiliations:** 1 Laboratory of Plant Germplasm Innovation and Utilization, School of Life Sciences, Liaocheng University, Liaocheng 252000, China; zhangjianling0520@126.com; 2 Laboratory of Molecular Biology of Tomato, Bioengineering College, Chongqing University, Chongqing 400030, China; qiaolixie@cqu.edu.cn (Q.X.); dtt@jsnu.edu.cn (T.D.); micy180605@163.com (J.L.); 3 Institute of Integrative Plant Biology, School of Life Science, Jiangsu Normal University, Xuzhou 221116, China

In the original publication [1], there was a mistake in Figure 8. A yeast two-hybrid assay of SlMBP21 and SlMADS1 with other sepal development-related proteins was conducted. In the original figure, the “positive control” and “negative control” were wrong. The four images of the “positive control” and “negative control” were the same as the four images for “pGBKT7-SlMBP21 & pGADT7-SlMBP21” and “pGBKT7-SlMBP21 & pGADT7-SlMADS1” due to the negligence of insufficient careful inspection. In addition, the authors performed the yeast two-hybrid assay again to verify the “positive control”, “negative control”, and the interactions of “pGBKT7-SlMBP21 & pGADT7-SlMBP21" and “pGBKT7-SlMBP21 & pGADT7-SlMADS1” (please see Figure S6). The corrected version of Figure 8 appears below. The authors state that the scientific conclusions are unaffected. This correction was approved by the Academic Editor. The original publication and the supplementary material have also been updated. 

## Figures and Tables

**Figure 8:**
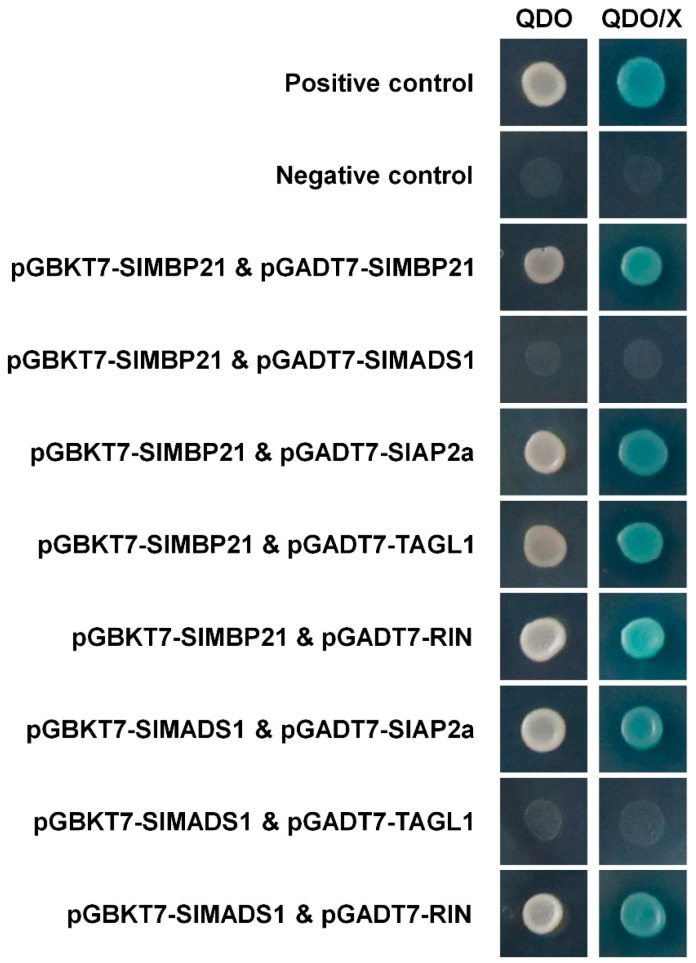
Yeast two-hybrid assay of SlMBP21 and SlMADS1 with other sepal development-related proteins. SlMBP21 could interact with SlMBP21, SlAP2a, TAGL1 and RIN but not with SlMADS1; SlMADS1 could interact with SlAP2a and RIN, but not with TAGL1, individually. QDO, SD medium lacking Trp, Leu, His, and adenine. QDO/X, SD medium lacking Trp, Leu, His, and adenine with X-α-Gal.

**Figure S6:**
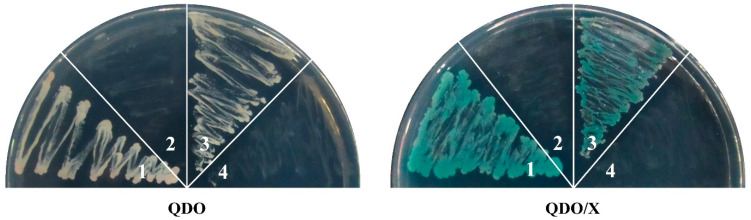
The “positive control”, “negative control”, and the interactions of “pGADT7-SlMBP21 & pGBKT7-SlMBP21” and “pGADT7-SlMBP21 & pGBKT7-SlMADS1”. (1) pGBKT7-53 and pGADT7-T (positive control); (2) pGBKT7-Lam and pGADT7-T (negative control); (3) pGADT7-SlMBP21 and pGBKT7-SlMBP21; (4) pGBKT7-SlMBP21 and pGADT7-SlMADS1; QDO indicates SD medium lacking Trp, Leu, His, and adenine. QDO/X indicates SD medium lacking Trp, Leu, His, and adenine with X-α-Gal.
