# Supplementary material for: Two SEPALLATA MADS-Box Genes, SlMBP21 and SlMADS1, Have Cooperative Functions Required for Sepal Development in Tomato
Source: Int J Mol Sci. 2024 Feb 20;25(5):2489. doi: 10.3390/ijms25052489 (PMC10931843; doi:10.3390/ijms25052489)
Supplement: Supplementary file 1 [file ijms-25-02489-s001.zip › Supplementary files.pdf]

Supplementary information

Two SEPALLATA MADS-box genes, *SlMBP21* and *SlMADS1***,** have cooperative functions required for sepal deveopment in tomato

Jianling Zhang[1, 2], Zongli Hu[1]*, Qiaoli Xie[1] ,Tingting Dong[1, 3], Jing Li[1], Changguang Liao[1], Guoping Chen[1]*

Jianling Zhang[1, 2], Zongli Hu[2]*, Qiaoli Xie[2] ,Tingting Dong[2, 3], Jing Li[2], Guoping Chen[2]*

1, Laboratory of plant germplasm innovation and utilization, School of Life Sciences, Liaocheng University, Liaocheng, Shandong Province, China.
2, Laboratory of molecular biology of tomato, Bioengineering College, Chongqing University, Chongqing, People's Republic of China;
3, College of Life Science, Jiangsu Normal University, Xuzhou, Jiangsu Province, People's Republic of China;

* **Corresponding author:** Zongli Hu, Guoping Chen

Guoping Chen: Room 523, Bioengineering College, Chongqing University, Campus B, 174 Shapingba Main Street, Chongqing 400030, P.R. China; **Tel:** 00862365112674; **Fax:** 00862365112674; **E-mail:** chenguoping@cqu.edu.cn

Table S1. Specific primer sequences used for *SlMBP21* and *SlMADS1* gene amplification and cloning procedures.

Primer Name	Primer Sequence (5'–3')	Application
SlMBP21-i	CGGGGTACCAAGCTTAATGGAGGACAAACAATGGAATAC<br>CCGCTCGAGTCTAGAGTCATAGTGCATAGTTGAGATGGG	To establish *SlMBP21* RNAi lines; add *Kpn*I+ *Hind* III and *Xho*I +*Xba*I site underlined, respectively
SlMBP21-1(p)-F	AAAACTGCAGAATGGAGGACAAACAATGGAATAC	To amplify *SlMBP21* fragment of *SlMBP21-SlMADS1*-RNAi vector; add *Pst*I and *Xba*I site underlined, respectively
SlMBP21-1(X)-R	TGCTCTAGAGTCATAGTGCATAGTTGAGATGGG	
SlMADS1-1(X)-F	TGCTCTAGAGATTACTCCGTAGAAA	To amplify *SlMADS1* fragment of *SlMBP21-SlMADS1*-RNAi vector; add *Xba*I and *Bam*HI site underlined, respectively
SlMADS1-1(B)-R	CGCGGATCCCAATGATACAAAAAATAC	
SlMBP21-2(K, H)-F	CGGGGTACCAAGCTTAATGGAGGACAAACAATGGAATAC	To amplify *SlMBP21-SlMADS1* fragments of the *SlMBP21-SlMADS1*-RNAi vector; add *Kpn*I+ *Hind* III and *Xba*I + *Bam*HI site underlined, respectively
SlMADS1-2(X, B)-R	CCGCTCGAGGGATCCCAATGATACAAAAAATAC	
SlMBP21-full	AGAAAAAGTGTGTTAACCTAGAGTGG<br>ATGTCATAGTGCATAGTTGAGATGG	Full-length amplification of *SlMBP21*
SlMADS1-full	AATTGAAGATTGATTTCTCAATGGG<br>GATCGCCGCCATAATTCCTG	Full-length amplification of *SlMADS1*
SlMBP21(Y2H)	CGCGGATCCTATGGGAAGAGGAAGAGTAGAACTA<br>AAAACTGCAGTTAGAGCATCCACCCTGGA	Construction of yeast two-hybrid vector
SlMADS1 (Y2H)	CCGGAATTCATGGGAAGAGGAAGAGTTGAG<br>CGCGGATCCTTAAAGCATCCATCCATGAATA	
SlAP2a(Y2H)	CCGGAATTCATGTGGAATTTAAATGATTCCCC<br>CGCGGATCCTCAAGGTCTCATAAAATAATGATGGA	
TAGL1(Y2H)	CCGGAATTCATGGTTTTTCCTATTAATCAGG<br>CGCGGATCCTCAGACAAGCTGGAGAGGAG	
RIN(Y2H)	CCGGAATTCATGGGTAGAGGGAAAGTAGAATTG<br>CGCGGATCCTCAAAGCATCCATCCAGGTAC	
NPT II	CTCAGAAGAACTCGTCAAGAAGG<br>GACTGGGCACAACAGACAATC	Positive transgenic plants detection

Table S2. Specific primer sequences used for qRT-PCR analysis

Primer Name	Primer Sequence (5'–3')	Product (bp)
SlCAC	CCTCCGTTGTGATGTAACTGG<br>ATTGGTGGAAAGTAACATCATCG	173 bp
q-SlMBP21	GTTAGATCAAAAAAGACTCAATCTATGCT<br>TGTATTCCATTGTTTGTCCTCCAT	172 bp
q-SlMADS1	CCTCCAACGATCTCAGAGAAACTT<br>CTGATCCAGCATGAATTGTGTCTT	139 bp
q-PEI	CGATGTCTATGGGACGGTTGA<br>ACAACAGGAACTAAATCCGATGC	198 bp
q-SlARP	GCTGGAAGATAAGGAATACTTCAG<br>GCAGGGCATTAGACAAGTCAC	218 bp
q-SlAR-1	TCTCACCCTAACGCTCCTACTG<br>CAAGTCCCCAACTCGCATAG	152 bp
q-SlACO1	ACAAACAGACGGGACACGAA<br>CTCTTTGGCTTGAAACTTGA	181 bp
q-ABP19a	GGTATTTCCACAAGGGTTACTGC<br>GGTGGCTGCGACAAGTTCA	154 bp
q-SlERF1	TTTTAGTATCGGATGGACG<br>GGCGGAGAAACAGAAGTA	102 bp
q-IAA3	AAAGTTATCAAGAGCTACTCAAGGC<br>CAACAAGCATCCAATCACCAT	138 bp
q-IAA9	TCTACTGGCTTCTTCAACTTC<br>CAGATAGACCCATATAGTTTCG	83 bp
q-SlAP2a	GAATGTACTGATAATGCAACGGACC<br>GCTGCTCGGAGTCTGAACCTTA	171 bp
q-SlCMB1	TGAGCGTCAACTGGATTCATCTT<br>CCCTCTGACTGAGCAGGTTGTT	213 bp
q-SlACS6	TGATCCTGGTGATGCATTTCTAGTTC<br>CTTCTTCTAAGGCTTCTTTTGTTACC	146 bp
q-GOBLET	CTGAACTTGACTGTATGTGGAGC<br>GAACGTTTACAACAAAGTGACAAT	158 bp
q-TAGL1	CGCAATAACTCCCTGCCTGTA<br>GAAGATGAAGAGCCTTGACCC	143 bp

```
-2800 TGAGGGATATATCAATTACTTTTTTCTTTTGAAAACAAATTTGGAGCACTTATTCAAATTTGAATCCAACTCAAAGTTGAATTTGAAAAATTTATAAC
-2702 AAAATATTAATTTTAAAATAATGAAAAAGTATATCATGATTCTCTTTTAATTTATTATGTCCAAAATTAGTTTTCCCCTTTCATGAAAAGTTGTAATTT
          ERE          ERE
-2603 TAATGAAAAATTGTAACTTTTATAAAAAGTTGCGATTTTTATGAAAGGTTGTCACTTTTATGAAAGGTTATGACGTTTACGAAAGATTGTAACCTTTT
-2505 CGGAGGGTTGTGACTTTTTTAAAGAGTTGCTACCTTTCCAATAGGTTATAATAAACAGTTGTTCACACTACCCTTTGTTGTTTATAAATAGAGGAATTT
-2406 TCTCTCATTATAAAGCAACAAAAATTCTGAATTTCTTCTTCTTCTTCTAAACAATTAAATATTCGTGTACTTTGTTCTTGTAGAGTGACTACTGAGACA
-2307 ATTGTTTTTGTATCAATACATTTGTGAAGAAAATCGTCCCATCCTGAGAGGATTTAATTCTTTAAACCTTAGGTACTAGTTAAGAATTATTTTTCATTA
-2208 GGAGATACTGTGTATTCAGTGAACATGATTTTTTTCCTTTTTATTATTCTATTTTTACATATCCTAATTTTTTTTACGGTCATTAATTTATGCTTATGAT
-2108 ATTTATATGTTTTAAATTCTATTATTTATGTTTCTGCAAAGTTATTTAACATGTACACATATAATAAGTAAATATATTCATTATGCATAAAATAATTAT
-2009 TGTACTCAGTTTCAAGAATTTATGTTCTTACATATTTATCTATCTATCTATCTATCTATCTATCTATCTATCTATCTATATATATATATATATATA
-1909 TATATGAAGTCTCCCACTTATCGGTAAGTGTAATTTCTTTTATTTTTTTTAATCTATTAACAAATTTTTAAATTTTTCCCTGAGCCATAACAATTGTATA
-1809 ATTTAAATATGTTCTTTTTCTTACGAAATGTCATAACAATTATTCTCTTCTTTATTTTTATTTTAATATTGATTAAAGAAATAATAATTTATTATTTTCT
-1709 CAATAATTCATTTATTAGAAATTCGTGAGTTATGTGATAATGATGAATAATGAAATTTATGTCAAAATATTATTAATATTTTGTATAATTATTTTTTCT
-1610 ATAATTTTTAGTGTATGAATATTAAAATATTAATAAAGGATTATTTGCAAGTTAATAAGAAAAAGACAGCTCGTAAGGGAAGCATCGACACGTTGGT
-1513 AATTAAACACTGGCGATTGTACTATTACGTTGATGTTCGTGTCCAAAAGGTAATACGATGTATCAATGTGAAACATTAATTTACCCAAAACCACAAA
                                         P-box
-1416 AAGGACCTACACTTATTACATATAGGGTAACTCCATGTACACCTATATATATACATATAATAATAAAATAAATAATATACATTCATAAATAATTGCAT
-1318 ATATATGTAGAGACACACTTTTTAAGGTGTATAAAAATATAACTTTTTAGAGTTCAGTTCTCAGGTGAAATTTATTAGTATCTGCTCTATCATTATTAA
-1219 TAGGTCTATGTTTGAGAACTATCACCAAGTTGATTGGGCTATTTTAGAAGGGTAGAGGTTTGACACAAAAGTTAATTTAGATAATATTTCTAATTGAT
-1121 GAATATAAGAAATATGTTTTTATTAATCGTACAGATACTTATAGTTTTACTTAATTTTGCTATTAGATTGATTTGTACAATTCGTCTTATAAGTATAAA
-1022 ATTTAAAATTTGTATAATTTTATTTTTGATTATATAAATCAAGAATTTTATGTATGTTTGTCCTAACTATTTGTATATAATTTATAAAAAAATTGATAA
               ERE
-923  TAAATTGTCTTGTTTGTATACTGAGAAACGAAATATACAAACAAATTTCTGAAAAAATTCTGACCGTATAAATACAGAATTTATATATATTTAACGTA
-825  TTTATATATTCGCAAGTGAAACTTATAAGCAGACATAAATATGCATACCAACATAAATATACAAACCTCAATCTTAATAGCAAATAAATATAAATAT
-728  AAAACACAATTATCGAAACTATAAGTCTAAAATCTTAATATATCTATTTTATTTGCTATTAGTAAGATCATGAGGGGGAGGCAGATGTGAGTTATGA
-631  GGATTCACTAGCTTTGACTAAATTGTTAAATAGTTAATAAGTATACGTAACAAATTTGTGTACTTAGTAGGCGTTTAATTATAGAACATTTTTTTAAG
-533  AAAATAAAAATTTCTTTATTTAAGTTTTTCAAAAAAAAAATCATCTCTTAAAATTTGTAAAAGAATTCATATTTGACGTGAAAAGATTATTACGTCAT
                                                                                          TGA-box
-435  ATCAGGGAATTAAAGAATAACCAGTTTAAATTATATTATTGTTTATTTATTGACTAGCGGATATTTATTTAATTATATTCTCAGTATTATTTTATATGA
-336  CATCATTTTTTTAGTTGATCTCAAAAAAAATGTTACCTTACTTCACACAAATATTCAAGATTTATATTAAACTACAAGTTTCCAAAAAATAAAAATAAT
-239  TCTTAAAAATAAAAAATGTGTCACATAAAATGAAACATATATATTTAAACATTTATATGTGACTTATCAATTTGAGTTGGGTTATATGAAAGTGACA
-141  AACGATTAGATAGGGCATAACAGTGCTTCTCTCTCTTTCCATTAAATTTGGTAGTCCACATACAGAGAGAGTAGAAAAAGTGTGTTAACCTAGAGTG
-44   GAATAATAGTGTTTTTCTTGTTAGAGACAAGAAAAGAGAGAAAAATGGGAAGAGGAAGAGTAGAACTAAAGAGAATAGAGAACAAAATAAACAG
```

Figure S1. Promoter analysis of *SlMBP21* gene.

Promoter sequence (2800 bp regions upstream the 5′ end of the predicted ORF) of *SlMBP21* gene was extracted from SGN database and searched against the promoter database plant CARE (http://bioinformatics.psb.ugent.be/webtools/plantcare/html/), three ethylene-responsive elements (ERE motif), one gibberellin-responsive elements (P-box) and one auxin-responsive element (TGA-box) are found in *SlMBP21* promoter region.

```
-2800 CATAGGTTTACTCGTAACTAATCGGTGTATTACTTAGGAAAGTGCTGAATGATAAAAAAAATTGGTTAGAATTTGGCCAAAAAATTAAAAAAACTAC
-2703 TCCTTTCTTTCATTTTATTTGTCATGTGAAATTTTTCGAAAGTCAATTTAACTAATTTTTAAAATTAAATTAGACTACATAAATTTAATATCTTAAATA
                                                         ERE
-2604 AAAAATAAAGATATTATATAAAAAAAACACAATAAATAGCAAATTTCTGTATATTAATTTGATTAAAATTACATCTTAAAATGTCAGTCAAAATTTT
-2507 TATAGGTTAAATTTAAAAATAGAAATTATGATAATTAAAAATAGACGAAGAGACTATGATATTTTTTCAATTTTCGATTAAACTAATCTTCTTTTGAT
-2409 TTGTAGTTAAAATATAAAAGTAAAAAGCTAAATAACATAAAATATCATTGTGCCCAAAAATATACTAATATTACAACATTGGGAGGGGAAATATTA
-2313 GGTATGAAAAAAAAAGGTACCAATAAAGTATCGATACGGAGACGGAGAGAAAGGGTAAAAGGTTCACTGTTTAAGGTTGTTGTTCATGTCCAATAG
-2217 ATATTACGATGTGCTAAAACATTAATCTACACAAAAAGTTAAGTATCTGAAAAGTACAAATTTACTATTTTTTCTCAGTTCAATAATAGTTCTTATAA
-2119 TCGACGGACTTTAAATACTTTTTAATAAATTAGGAATAGAAGAGTAATTTTATTATATAATTTTTTAATAATATTTTGAAAAATATAACAGAAATTAT
-2021 TATAATTAATAATAATAATAATAAATTAAAAACTTAATATAAAATTATTTATTAATTTTATAAAGTAAAAAAATATTGATAAACATCTCAATATAATA
-1923 TAGTGAATAATTGTTATTAAATCAAGGGATATTATACACTTACTAGCTAACTCTAAAAATCTATTACTATAAAAGAAGGGTGCACTACACAAATATC
-1826 TATATTTGTAATTTATTGACATAAAATTGCATGTACTGATGGTATAAAATTTATTTAACAAACTTATCATGTACTACTCACTGTATGTTAATAGAGTGA
-1727 GTGTGGGATACATTTTGGGTATGAAGGACGAGTTGACTCGATTAAAAATAAAGATAATTTGTTTTTACACTAGTTCTACTTTATAGGATATAATTTG
         TATC-box
-1630 ATAATGAATTAAAATAAATAATTAATAATTACAAAGTGTGAAATGAGAATTTTAAAAATAAAATAAAACTGAATATATTTTTTTTAATAACTAAAAA
                                                  ERE
-1533 GAAAAATGAGACATAAAAATCAGAATAGAAGAAGTTATGTACATTTGTTAGGTGTGATCCATAGGATGCTAATAACTTGGTTGATTTGACAAAAGG
                                                                                              P-box
-1437 AAATTGTTGTGTTATTTAGAAAGAAAAAGAAAATTCTAATAATACTCTAATTATATTTGAAGTGAAATTAATTTTTCCCTTAAAATGACATAATTAGA
-1339 TATTTTCACAATATTCATCATTTTCGTTCAAATTATTTTCTGTCTTCATCAATCAGCAACCGTCCAATATTCCTTCATTAATTAAAAACCGTCCAATAA
-1240 GCCAACACAATCCTTTTATTTCACAGTTTAATAATCTTTCAACATTTCTCCATCATTCAAAATTCTATACATTTACTTAAATAAATAATTTTCTACTACT
-1140 TCTAACACTCAAAAAATTTGCTAGTAATGTTTCTATTTATATATAATAGCATTGCAAAACCTAATTTATCTTGAAAAAGAGAATATATTCCTAGTGAA
-1042 TTATTAAAACAAAAAAAGCCTATTATAATATGAGTTAAATAAATACCCAAATCAATTTTGATTTCCTACCACTTTAAATTATTCTCTCGACTTTGAAC
-944  AACGCGTCAGTCGTAATAGATTAATTTTTATGCTAATTTTTTTTACTTTCAACATAAAAATTATATTTTAGGAAAGAATAACGAACGAGGTATTATGA
-846  TACTCTTCGTTTTGTCGAGACCTACTTTGCTCTTTGGTTTGATAATTGTACGTGCTGAGTTCGAACAATGTTTGGAGATATTAAGTCGCTTGCAATATT
-747  AACATACATAAAATAACTCGATCATTTGAATAATTAGAAGTAGCGCTTTATTAGCTTGACAATTGTTGAGGATACCATTAAGGGAAAAAAGACAAAT
-650  ATAACCCCCAAGTATCGTTTTTGTCGTAAGCAAATACTTTTCGTCATACTTTTGACATATTGGTGCCCTGCCACCCAGAAATAGAACATATATACCCT
-552  TTAAACTAACAGACATACAATTGTCATAATCTTATCCACCGACTTGTCATTTTCATATTGAATATATGATCAATGAATATGATTGTGTCACGTGTCTCT
-453  GTTCAGTCTTATTAGAGTAAAGGATATATATGCTCTAGTTTTTGGACGGTGGGGACACCAATATCGTCCCAAAAGTATGACGGAAGATATCTGCATA
-356  TTATTAATGATAATTCAAAGATATATTTGTTTTTTTCCCCCTATAATTAAATAACAAAAATGTACTATCTCTATCGATATAAACTTTTAAATGACATGA
-257  TCGCACGTTTCTAACGTGATATTAATAGTTAGATGCTCTTTGTTTTAACTTTTGGGTTTTTTAGTGGGGGTGTAAATGGAAAGGTACCAAAGATAGAG
-159  AAAGTCCCAGGGTTTCCATATTTCCAACTCTCTCTTTCTTTCATTTATTTTAATTTGTACCTTTTGTTATCCAAAAGTAGTTGAGCTTGTAACCAATAA
                   ERE                                     P-box
-60   TCCCCCCTTCTTTTCTTATTTTTTTTAAAATCTCTGAAAAAAATTGAAGATTGATTTCTCAATGGGAAGAGGAAGAGTTGAGCTTAAGAGAATAGAAA
```

Figure S2. Promoter analysis of *SlMADS1* gene.

Promoter sequence (2800 bp regions upstream the 5′ end of the predicted ORF) of *SlMADS1* gene was extracted from SGN database and searched against the promoter database plant CARE (http://bioinformatics.psb.ugent.be/webtools/plantcare/html/), three ethylene-responsive elements (ERE motif) and three gibberellin-responsive elements (two P-Box and one TATC-box) are found in *SlMADS1* promoter region.

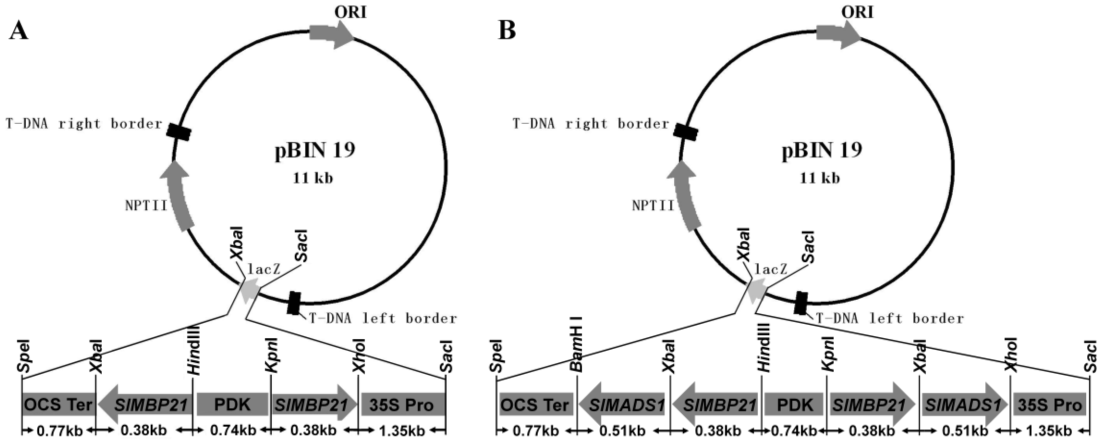

Figure S3. The construction of *SlMBP21*-RNAi and *SlMBP21-SlMADS1*-RNAi vectors

(A). Hairpin construct of the *SlMBP21* gene for double-stranded RNAi vector. The *SlMBP21* gene-specific sequence in the antisense and sense orientations were linked with a PDK gene fragment and as a transcriptional unit for hairpin RNA expression which premoted by the CaMV 35S promoter and terminated by the OCS terminator. Among which, *Spe*I and *Xba*I are isocaudamers. (B). Hairpin construct of double genes, *SlMBP21* and *SlMADS1*, for double-stranded RNAi vector.

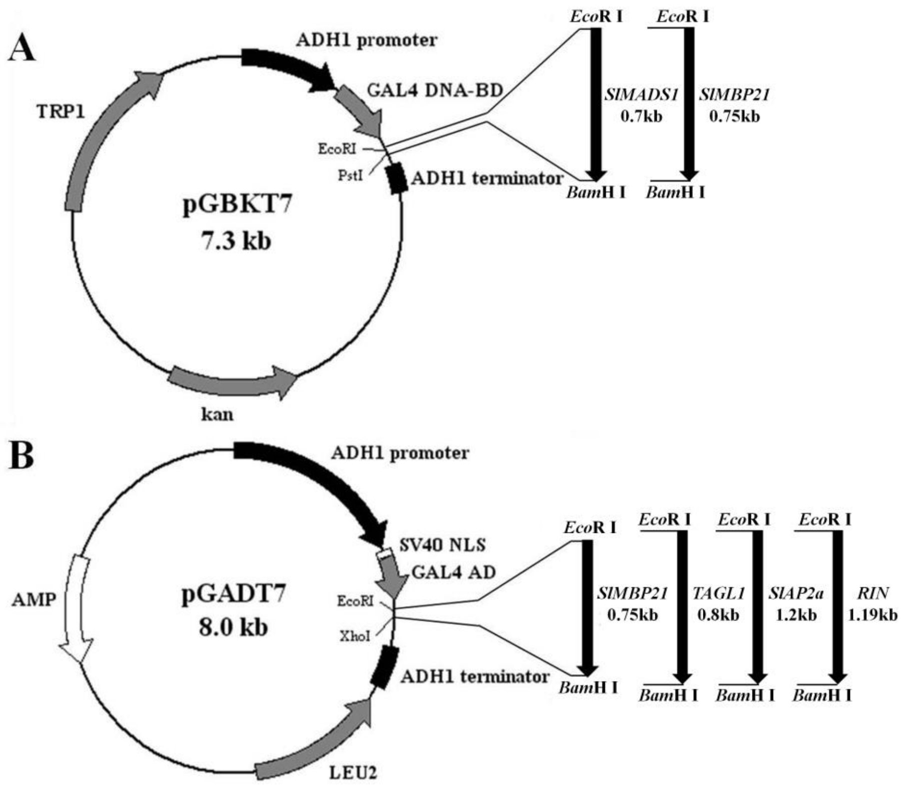

Figure S4. Construct of *SlMBP21*, *SlMADS1*, *SlAP2a, TAG1* and *RIN* gene for the yeast two-hybrid vector.

(A). The ORFs of *SlMBP21* and *SlMADS1* were cloned into pGBKT7 bait vector to obtain the vector pGBKT7-*SlMBP21* and pGBKT7-*SlMADS1*, respectively. (B). The ORFs of *SlMBP21*, *SlAP2a*, *TAG1* and *RIN* were cloned into pGADT7 prey vector to obtain the vector pGADT7-*SlCMB1*, pGADT7-*SlMBP21*, pGADT7-*MC*, pGADT7-*TAGL1* and pGADT7-RIN.

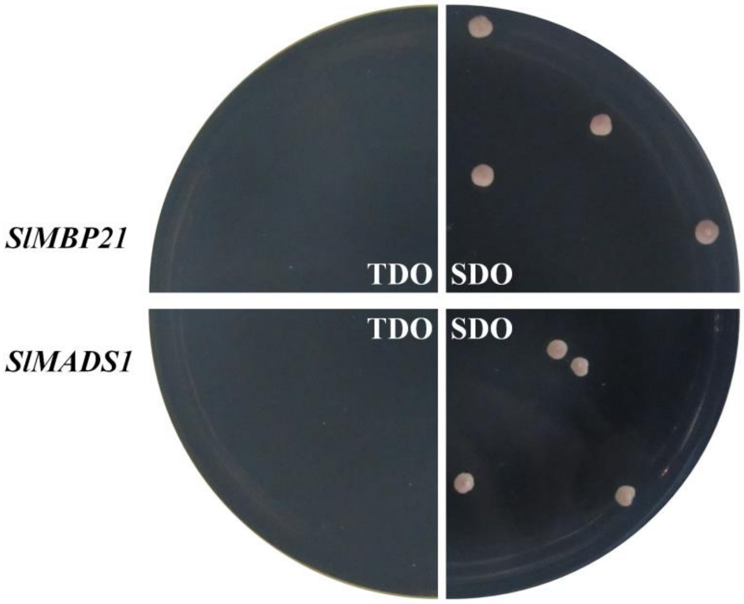

Figure S5. Self-activation assay of pGBKT7-*SlMBP21* and pGBKT7-*SlMADS1* in the yeast two-hybrid assay.

Yeasts with pGBKT7-*SlMBP21* and pGBKT7-*SlMADS1* were plate on SDO and TDO medium. All these two yeasts had no self activation, they can grow on the SDO medium but can not grow on the TDO medium, respectively. TDO, SD medium without Trp, His, Ade; SDO, SD medium without Trp.

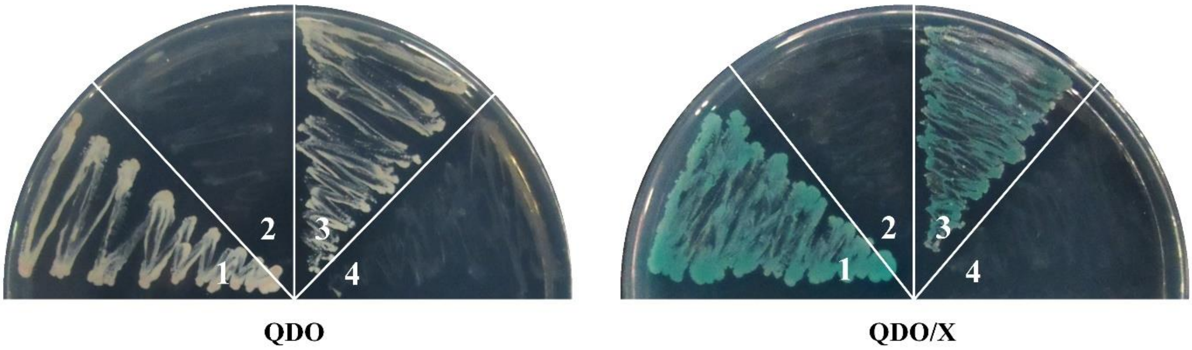

Figure S6. The “positive control”, “negative control”, and the interactions of “pGADT7-SlMBP21 & pGBKT7-SlMBP21” and “pGADT7-SlMBP21 & pGBKT7-SlMADS1”. (1) pGBKT7-53 and pGADT7-T (positive control); (2) pGBKT7-Lam and pGADT7-T (negative control); (3) pGADT7-SlMBP21 and pGBKT7-SlMBP21; (4) pGBKT7-SlMBP21 and pGADT7-SlMADS1; QDO indicates SD medium lacking Trp, Leu, His, and adenine. QDO/X indicates SD medium lacking Trp, Leu, His, and adenine with X-α-Gal.
