# Supplementary figures and images for: Two SEPALLATA MADS-Box Genes, SlMBP21 and SlMADS1, Have Cooperative Functions Required for Sepal Development in Tomato
Source: Int J Mol Sci. 2024 Feb 20;25(5):2489. doi: 10.3390/ijms25052489 (PMC10931843; doi:10.3390/ijms25052489)

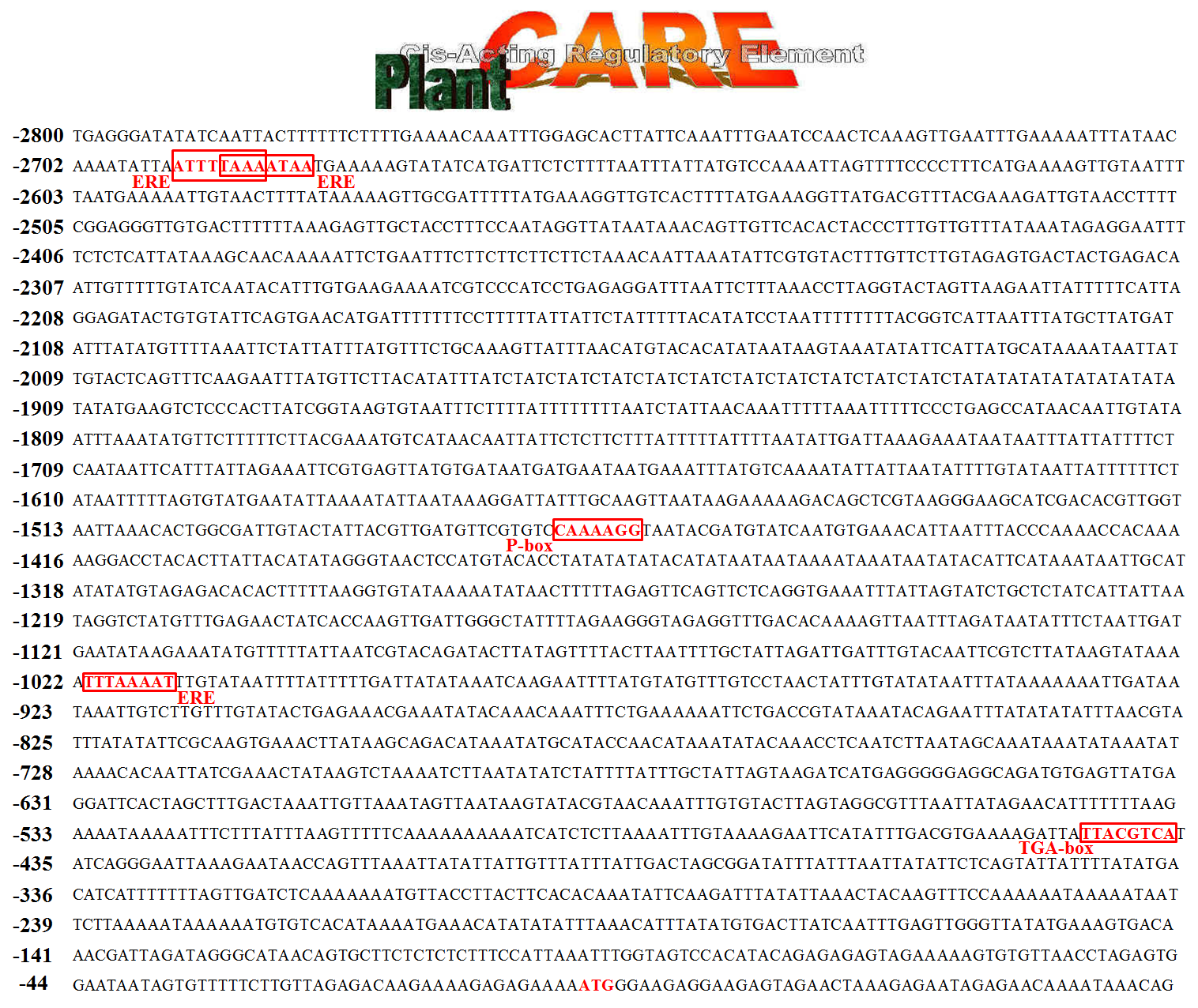

Supplement: Supplementary file 1 [file ijms-25-02489-s001.zip › Figure S1.tif]

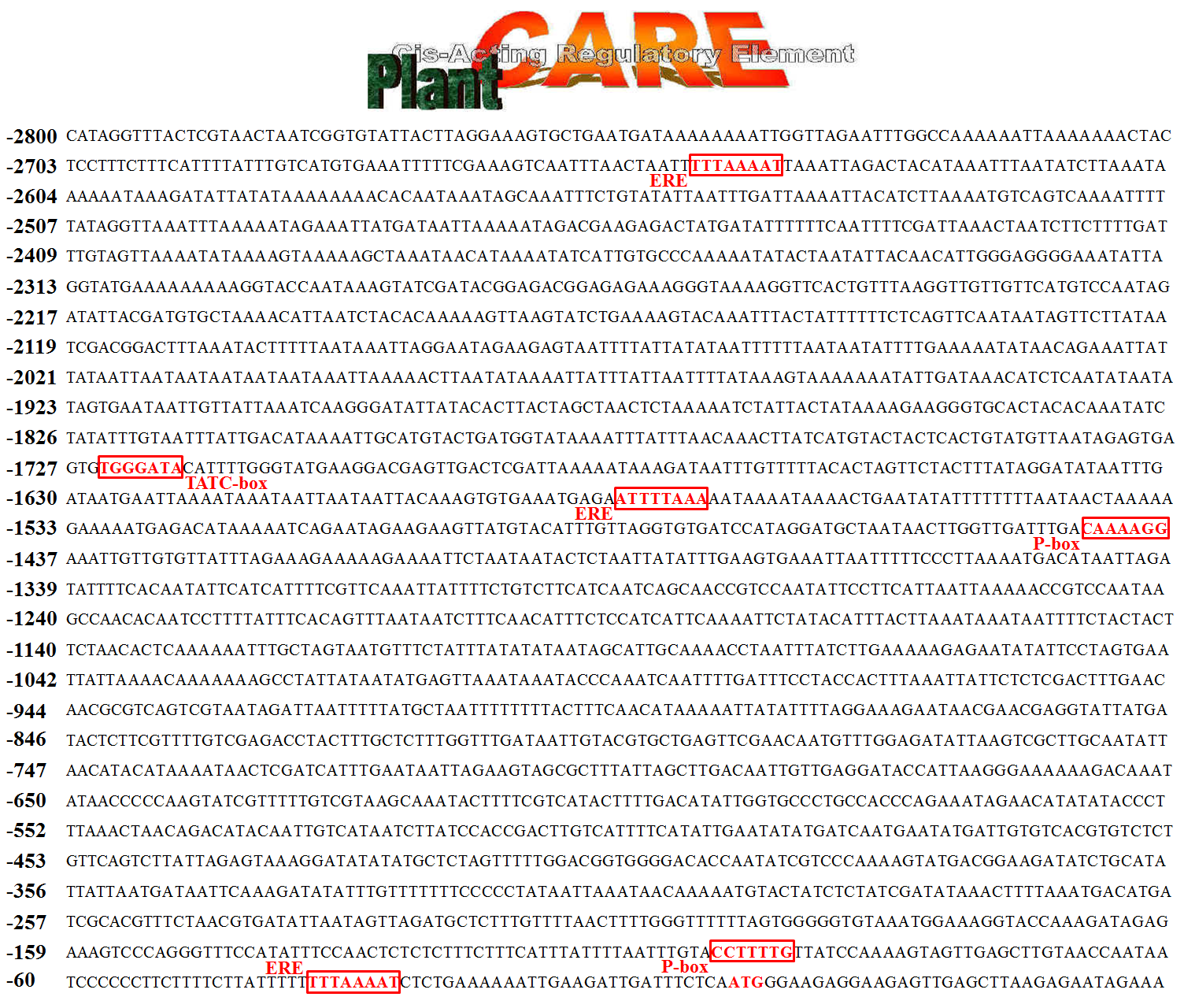

Supplement: Supplementary file 1 [file ijms-25-02489-s001.zip › Figure S2.tif]

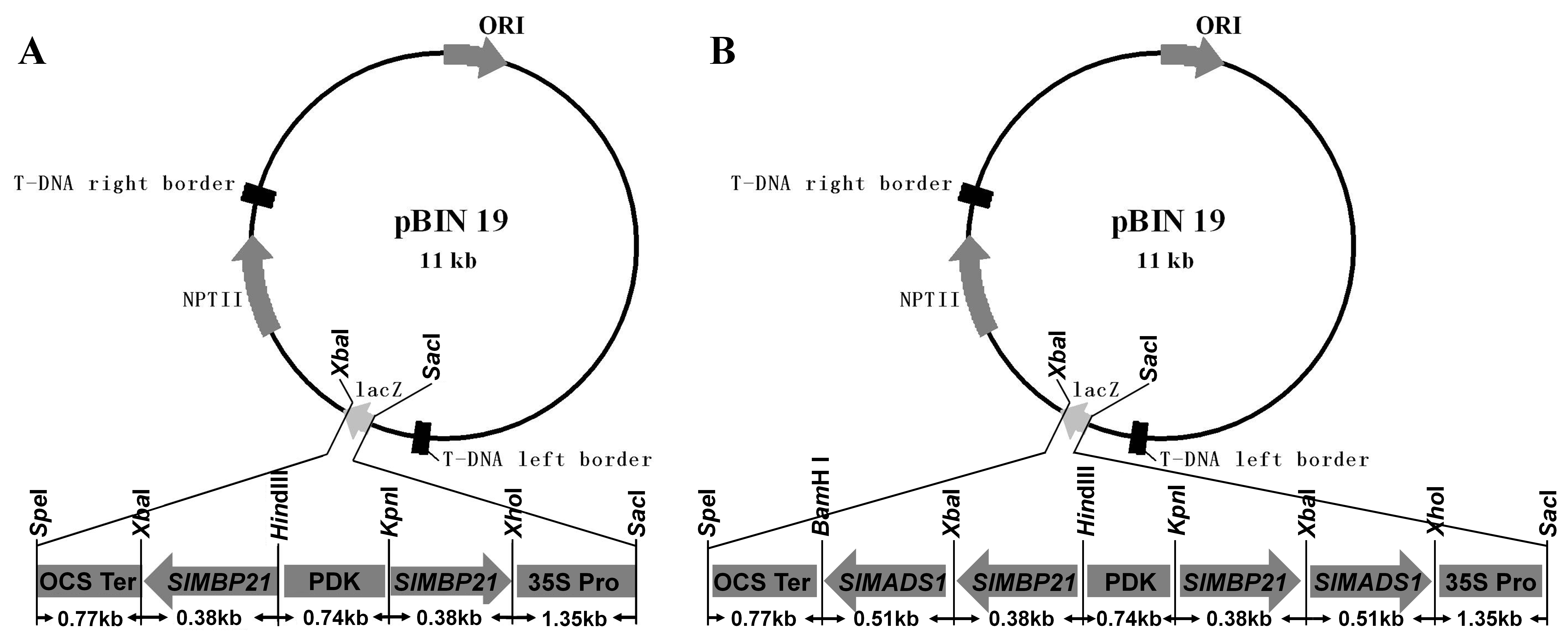

Supplement: Supplementary file 1 [file ijms-25-02489-s001.zip › Figure S3.tif]

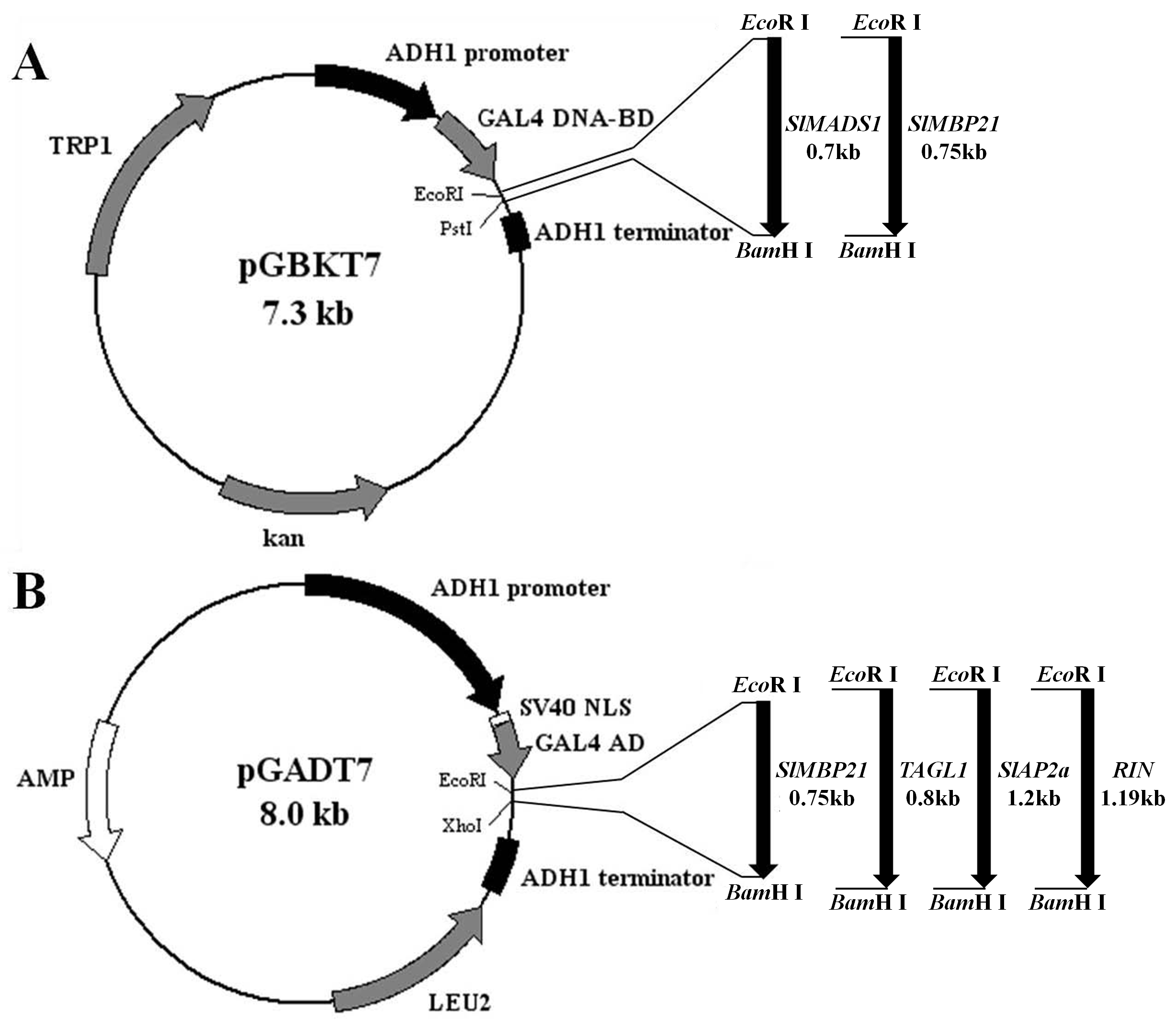

Supplement: Supplementary file 1 [file ijms-25-02489-s001.zip › Figure S4.tif]

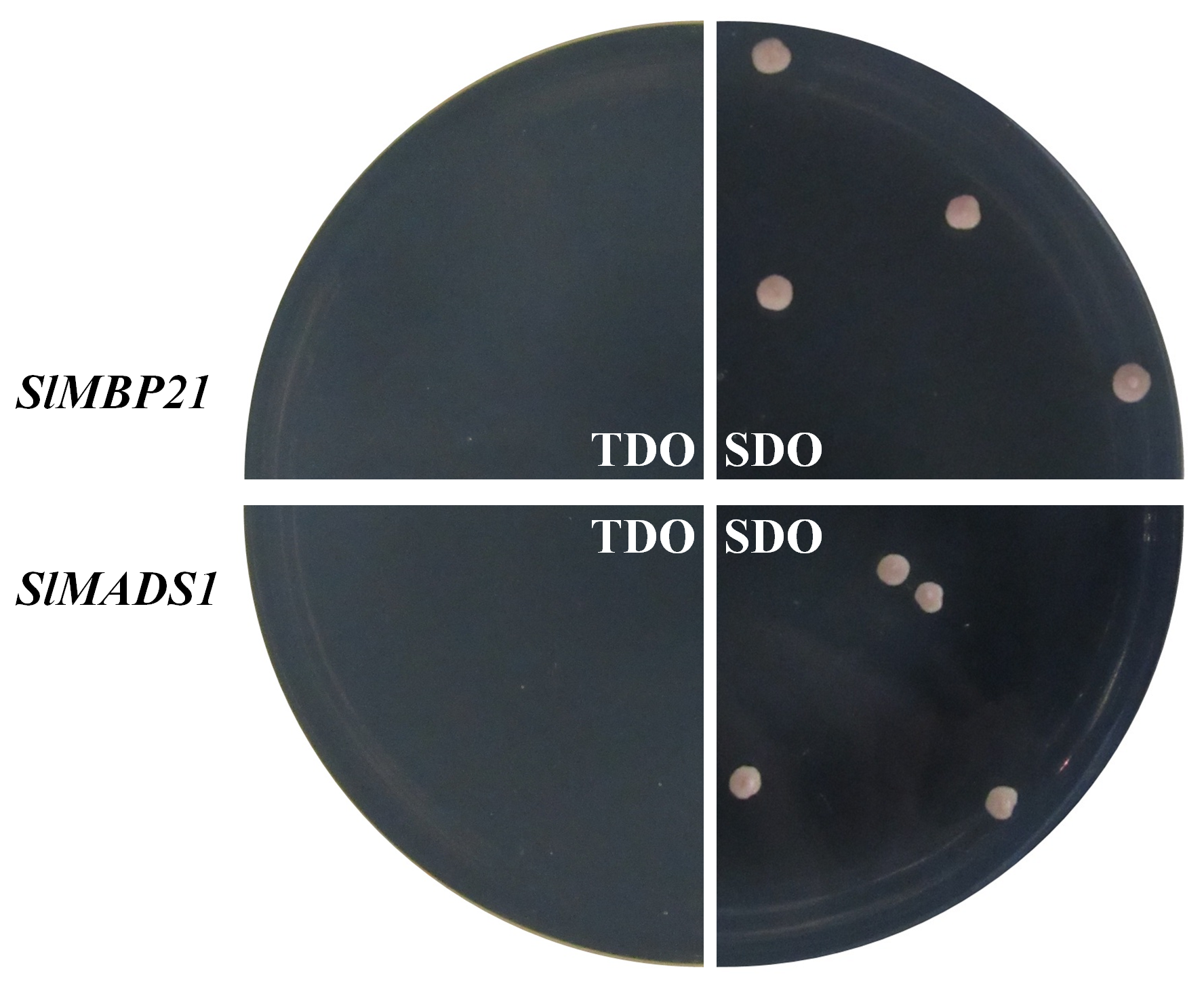

Supplement: Supplementary file 1 [file ijms-25-02489-s001.zip › Figure S5.tif]

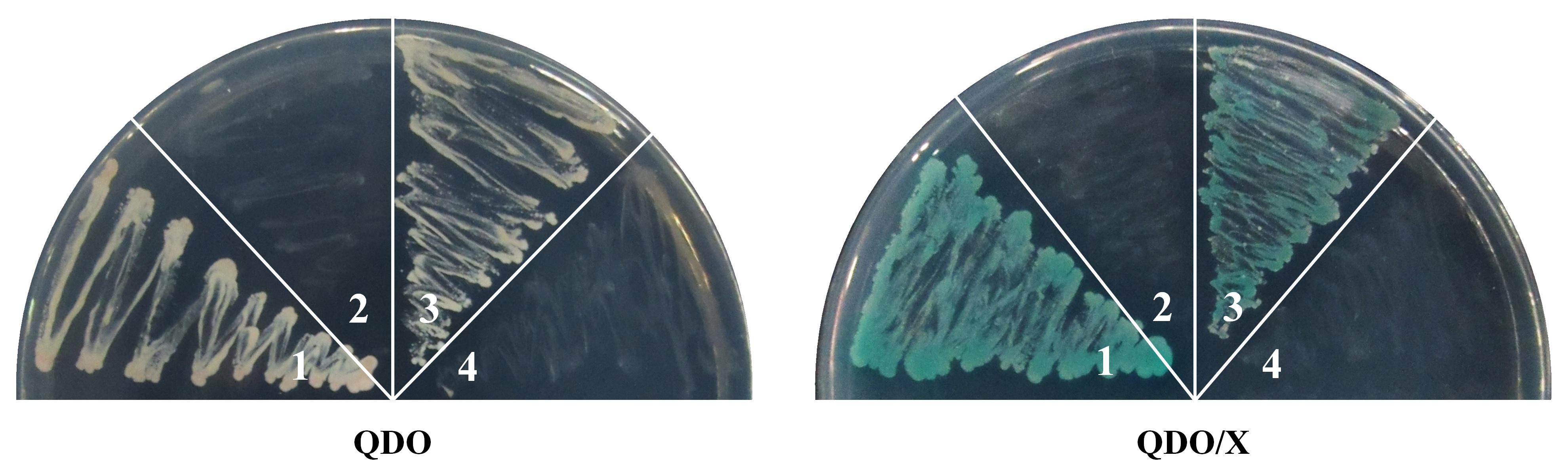

Supplement: Supplementary file 1 [file ijms-25-02489-s001.zip › Figure S6.tif]
